# Resistance Development to Bacteriophages Occurring during Bacteriophage Therapy

**DOI:** 10.3390/v10070351

**Published:** 2018-06-30

**Authors:** Frank Oechslin

**Affiliations:** Department of Fundamental Microbiology (DMF), University of Lausanne, CH-1015 Lausanne, Switzerland; frank.oechslin@gmail.com

**Keywords:** bacteriophage, phage, phage therapy, phage-resistance

## Abstract

Bacteriophage (phage) therapy, i.e., the use of viruses that infect bacteria as antimicrobial agents, is a promising alternative to conventional antibiotics. Indeed, resistance to antibiotics has become a major public health problem after decades of extensive usage. However, one of the main questions regarding phage therapy is the possible rapid emergence of phage-resistant bacterial variants, which could impede favourable treatment outcomes. Experimental data has shown that phage-resistant variants occurred in up to 80% of studies targeting the intestinal milieu and 50% of studies using sepsis models. Phage-resistant variants have also been observed in human studies, as described in three out of four clinical trials that recorded the emergence of phage resistance. On the other hand, recent animal studies suggest that bacterial mutations that confer phage-resistance may result in fitness costs in the resistant bacterium, which, in turn, could benefit the host. Thus, phage resistance should not be underestimated and efforts should be made to develop methodologies for monitoring and preventing it. Moreover, understanding and taking advantage of the resistance-induced fitness costs in bacterial pathogens is a potentially promising avenue.

## 1. Introduction

Antimicrobial resistance is a major public health problem that could possibly cause an estimated 10 million mortalities per year by 2050 [1]. For this reason, novel therapeutic strategies, beside traditional antibiotics, must be rapidly developed. One of these strategies is the use of bacteriophages (phages). Phages are nature’s most abundant bacterial predators. They can be used alone or in combination with antibiotics against difficult-to-treat infections. Phage therapy has been used since the 1920s in the Soviet Union, and is still currently used in ex-Soviet countries like Poland, Russia, and Georgia [2]. Phage therapy is currently being revisited as a potential alternative to antibiotics in Western countries. However, challenging issues still exist, such as selecting the most adequate phage(s) against a given infection, the risk of phage resistance development, the immune response to phages by the host, as well as novel regulatory requirements [3,4].

Bacteria can resist phage attack through different mechanisms, including spontaneous mutations, restriction modification systems, and adaptive immunity via the CRISPR-Cas system [5]. Spontaneous mutations are the main mechanisms driving both phage resistance and phage–bacterial coevolution [6]. Spontaneous mutations may confer phage resistance by modifying the structure of bacterial surface components that act as phage receptors and that also determine phage specificity. These include lipopolysaccharides (LPS), outer membrane proteins, cell wall teichoic acids, capsules, and other bacterial appendices, such as flagella, many of which may all be part of virulence factors (e.g., LPS) [7].

However, phage resistance may also induce trade-off costs. Phage-resistant bacteria may become less virulent in case of mutations in surface virulence factors, such as LPS [8]. Likewise, the maintenance of anti-viral defence systems, such as for DNA restriction-modification enzymes and CRISPR-Cas adaptive immunity, also has its own cost [9,10].

This review discusses the implications of the development of phage resistance in the perspective of implementing phage therapy. Phage resistance is first considered in the context of population and phage–bacterial evolutionary dynamics. It is then considered in the frame of experimental therapy (summarized in Table 1), in order to determine its role in treatment failure or salvage therapy strategies.

## 2. The Evolution Dynamics of Resistance

Phage–bacteria coevolution can be defined as a process of reciprocal adaptation and counter-adaptation between the phage and its bacterial host. It is an important driving force for the ecology and evolution of microbial communities [6]. Phage–bacteria interactions are mediated first by phages using their tail fibres to adsorb onto the bacterial surface, through a lock-key mechanism. Since the complete phage replication life cycle, namely the lytic cycle, relies on the killing and lysis of the host bacteria, a strong reciprocal selection pressure evolves toward increased infectivity on the side of the phage, and phage-resistance on the side of the bacterium [41]. Bacteria can evolve phage-resistance by de novo chromosomal mutations, as well as through an arsenal of antiviral mechanisms targeting virtually all steps of the phage life cycle (reviewed in [5]). For example, bacteria can prevent phage adsorption by modifying the structure of their surface phage receptors, or by hindering the access of the phage to the receptor through the production of an excess of the extracellular matrix, or even by producing competitive inhibitors [42,43,44].

The development of phage-resistant bacteria was already described almost a century ago in a seminal paper by Luria and Delbrück, who observed that the initial phage-induced lysis of a bacterial population was followed by bacterial regrowth, due to the selection of a phage-resistant sub-populations [45]. Phage-resistant bacterial variants that were already present in the initial bacterial culture (at a rate of ca. 10^−8^) were selected and led to the replacement of the entire culture with the resistant variant. This extreme situation leads to an evolutionary dead-end, for instance if the phage receptor is lost, and phage do not have the opportunity to develop a counter-resistance. A large number of studies concluded similarly on the absence of phage–bacterial coevolution following the emergence of phage-resistant bacteria (reviewed in [46]). This was the case in chemostat experiments using *Escherichia coli* infected with series of T or lambda phages. Phages could not interact anymore with the resistant *E. coli* variants [47]. However, phage-susceptible parent bacteria could still be recovered from sanctuary niches such as biofilms present on the chemostat’s walls. These survivors allowed maintaining low levels of phage persistence.

Other studies observed more persistent cycles of coevolution between phages and the host bacteria. This was the case for *E. coli* O157:H7 and phage PP01 in a continuous chemostat culture [48]. In this experiment, phages could coexist with phage-resistant variants and evolve different host ranges for the phage-escape bacterial mutants. Phage-resistance was associated with a dual bacterial population carrying either LPS alterations or OmpC surface protein silencing. Moreover, a third type of mucoid colony mutant emerged and could coexist with phages until the end of the experiment. Eventually, phage mutants with different host ranges also appeared.

One possible explanation was that none of the three phage-resistant bacterial mutants were completely immune to phage infection and, thus, entered a phage coevolution cycle permitting parallel phage and bacterial expansion and selection for phage variants with broader host range.

In other experiments, using lambda phages, an arms race was observed when using minimal media and maltose as the only carbon source [49]. Since the lambda phage uses the maltose outer membrane porin, LamB, as a bacterial receptor, decreasing LamB synthesis decreased phage susceptibility. However, this also decreased bacterial fitness in the presence of a lactose-only carbon source. In parallel, phages selected variants with increased LamB affinity or new variants able to infect via alternative receptors. Phages shifted from using the LamB to OmpF receptor through amino acid substitutions in their tail fibre J protein [50]. This mutual counter-selection process between the phage and the bacterium that enabled each of them to survive, without eliminating the other.

Further confirmation of the arms race came from observations with *Pseudomonas fluroescens* and the Podovirus Ф2 [51,52]. In this case, reciprocal evolution of infectivity and resistance was followed during >100 bacterial generations, with phages becoming more broadly infectious and bacteria more broadly resistant over time [41,52]. However, the arms race became progressively weaker, with increasing fitness cost due to generalist adaptive mutations on both sides [53].

A common paradigm in evolutionary biology is that evolution tends to maximize the adaptation ability (in this case phage-resistance) by allocating resources preferentially when they are limited. If resources are dedicated to phage-resistance, a fitness cost may be associated with a mutation conferring phage resistance that arises during coevolution, as demonstrated for the altered integrity of LPS and OmpC resulting in heterogeneous populations in the example of *E. coli* O157:H7 and phage PP01 [48], and altered maltose uptake with porin LamB mutation in the example of lambda phage and maltose restriction [49]. Numerous other examples exist regarding various phage–bacteria coevolution systems (see [54,55,56,57]). Environmental conditions can also greatly impact the coevolution of virulence and resistance by imposing limits to the arm race, as exemplified by the LamB example where maltose becomes a limiting factor [49]. This may result in different coevolution routes in natural settings from what is observed in the test tube. Such exogenous factors can include UV light-induced mutagenesis, which may induce phage-resistance mutations, but also cause additional mutations affecting in parallel [57]. Other examples include the influence of still or shaking culture conditions on population structures, which may influence particle collisions between phages and bacterial preys, or niche resource availability [58,59,60].

A recent work with *P. fluorescens* SBW25 showed that phage–bacterial interaction increased the bacterial mutation rates as well as its chance to adapt and survive both predation and altered environmental conditions. This indicates that phage-driven evolution may be ultimately beneficial for the bacteria [61].

Interestingly, while this phage-stimulated mutation rate of *P. fluorescens* SBW25 was observed in laboratory conditions [61], it was not observed for *P. fluorescens* communities living in the soil [62], most probably because selection for phage-resistance is higher in the soil than in vitro [63]. It is then assumed that, unlike in vitro coevolution that is characterized by the so-called arms race of increased resistance versus infectivity over time, coevolution in natural environments is largely driven by fluctuating selection, where cycles of phage-susceptible and phage-resistant bacterial populations intertwine with parent phages and evolved phages (mutant phages) that regain infectivity against the resistant bacteria [41].

## 3. Emergences of Phage Resistance in Animal Models

### 3.1. E. coli Diarrhoea in Cattle

Controlled studies on phage therapy and the emergence of phage resistance started in Western countries with a series of farm animal trials against experimental diarrhoea with enteropathogenic *E. coli* strains. In a first trial, oral phage therapy prevented *E. coli*-induced diarrhoea in colostrum-fed calves even when given 8 h after bacterial inoculation [14]. No phage-resistant variants were isolated from the calves. In another trial, phage therapy was administered at the onset of diarrhoeal symptoms, but resolved the intestinal symptoms in only half of the animals (14 out of 21 calves died). Phage-resistant variants were recovered from the small intestine of all calves failing clinical improvement. In parallel, the faecal bacterial content of 11 calves that responded to phage therapy was examined over a period of 14 days. *E. coli* titres progressively decreased over time and phage numbers increased during the first 48 h, therefore indicating phage replication. Phage-resistant bacteria emerged after 19 h, peaked at 2 days and decreased to undetectable levels after 10 days. Of note, these resistant mutants did not proliferate in the small intestine and did not cause diarrhoea when reinoculated to healthy colostrum-fed calves. The reduced virulence was associated with loss of the K antigen, which is known to be a virulence factor for enteropathogenic strains [64].

Prevention of diarrhoea by oral phages was also observed in piglets and lambs infected with *E. coli* [14]. Resistant variants were also isolated in the piglet faeces, but at relatively low rates. In contrast, no phage-resistant variants were observed in lambs. Similar results regarding the potential of phage therapy to prevent or treat *E. coli* diarrhoea were described in a second study done by Smith and Huggin [15]. This time, K-positive phage-resistant *E. coli* variants were isolated in addition to the already-observed K-negative variants. However, unlike the K-negative resistant variants, K-positive resistant variants were as virulent as the parent strain. These resistant variants were isolated only in vivo from the calves and not in vitro from a broth culture.

In another study in Holstein steers, phage therapy could reduce the average number of *E. coli* O157:H7 CFU in the faeces compared to controls, although it did not eliminate the bacteria from the majority of animals [16]. No phage-resistant *E. coli* O157:H7 mutants were observed.

Thus, phage-resistant variants are readily selected during phage therapy in vivo, but their pathological significance is unclear.

### 3.2. E. coli and Enterococcus faecalis Intestinal Colonization in Mice

Three studies using mouse models of *E. coli* intestinal colonization have also investigated the effect of phage therapy on the emergence of phage-resistance. In the first study, *E. coli* O157:H7 intestinal carriage could be eliminated within 48 h using three repeated phage oral doses. No resistant colonies were recovered and untreated control mice remained culture-positive for 10 days [16].

In the second study, an oral cocktail composed of three different phages was administered to mice colonized with enteroaggregative *E. coli* O104:H4 [18]. The bacterial titres did not decrease as compared to controls as expected and remained stable for 21 days, although phages were observed to replicate continuously during the time of the experiment. In addition, bacteria recovered on day 21 were still susceptible to each of the bacteriophages present in the cocktail when tested separately.

The third murine gut colonization study was a long-term 240 day protocol using oral T4 phages. Phage-resistant *E. coli* emerged only on day 92 and resistant variants constituted 100% of bacterial colonies isolated from phage-treated mice [17]. In comparison, only 20% of untreated mice carried phage-resistant variants. Moreover, when phage therapy was stopped at day 92, the presence of phage-resistant *E. coli* persisted over the 240 experimental days. The mechanism of phage-resistance was not described.

In an additional work, Duerkop et al. inoculated germ-free mice with *E. faecalis* V583 before starting phage therapy, which was first administered by oral gavage 6 h after colonization followed by administration in drinking water [19]. Phage therapy slightly decreased faecal bacterial loads by three-fold after 24 h. However, the level of colonization was not different from control animals after 48 h, even if phages were added to the drinking water. While 100% of the *E. faecalis* isolates remained phage-susceptible after 6 h of treatment, only 15% were still susceptible at 24 h and 100% were resistant at two days. Sequencing of 20 resistant bacterial variants revealed that they all had various mutations in the integral membrane protein PIPef, which was observed to promote phage infection.

Finally, using a model of gnotobiotic mouse intestinal colonization, Reyes et al. analysed the impact of viral predation on a consortium of 14 human bacterial symbionts [65]. When the community was subjected to virus-like particles purified from the faecal microbiota of human healthy donors, changes in the relative bacterial abundance was observed, but not with heat-killed viral-particle preparations. Especially, *Bacillus caccae* bacterial communities were observed to first decrease after phage attack, although they recovered later on. Evidence that phage resistance occurred due to genetic changes, like acquisition of CRISPR elements, could not be observed. The authors hypothesized that resistance was more the result of the expansion of an unexposed fraction of the population that could be protected in intestine microhabitat.

### 3.3. Control of Poultry Pathogens

A large part of the phage therapy application to control pathogenic bacteria in animals has been done in poultry, in order to prevent *Salmonella* spp. and *Campylobacter* spp. gut colonization and infection. *Salmonella enterica* is one of the major causes of foodborne infection in humans due to its symptomless carriage by chickens [66]. Up to now, the use of phage therapy to control *S. enterica* in poultry could reduce, but not eliminate, the bacteria. Sklar and Joerger reported that phage therapy did not significantly decrease *S. enterica* intestinal carriage in three animal trials [20]. Although phage resistance was observed after treatment, the authors speculate that other factors, like the salmonella intracellular lifestyle, could have contributed to the therapeutic failure. Atterbury et al. observed that low phage concentrations were not efficient in significantly decreasing bacterial loads [21]. They proposed that the low salmonella concentration in the chicken gut, associated with the complexity of the intestinal milieu—including physicochemical conditions, such as viscosity—was not suitable for phage amplification. Increasing the phage titre of phage preparations increase efficacy, but did not result in bacterial eradication. Interestingly, phage-resistance rates were higher following higher phage titres, indicating phage–bacteria interactions took place. Moreover, phage-resistant bacteria were not hampered in their ability to colonize the gut, and they often reverted to the susceptible parent phenotype. These observations were further confirmed by Carvalho et al., including the fact that phage-resistant mutants were able to colonize the gut, possibly by quick reversion to the parent phenotype [13].

Carrillo et al. reported somewhat analogous results with *Campylobacter jejuni* [11]. Phage treatment resulted in a decrease in the bacterial load ranging from 0.5 to 5 log CFU/g depending on the amount of phage and time of administration. As for *S. enterica*, certain phage-resistant isolates were observed to have decreased colonization abilities, but quickly recovered by reverting to the phage sensitive phenotype. Interestingly, phage-resistance in *C. jejuni* entailed a large (90 kb) genomic inversion at Mu-like prophage DNA sequences. The resulting cells demonstrated resistance to virulent phages, inefficient gut colonization and production of infectious bacteriophage CampMu particles [12]. Recovering gut colonization capability was associated with re-inversion of the DNA fragment. The observation revealed unprecedented phase-variation resistance mechanisms that could also occur in other bacteria. Later observations by Sørensen et al. suggested that phage-resistance phase variation was associated with excess capsular polysaccharide production in the case of chicken co-infection with *C. jejuni* and phage F336 [67]. However, resistant variants had kept their gut colonization capability.

Taken together, these different studies raise the question of the selection of phage-resistance in the gut environment and its implication for phage therapy. Indeed, the complexity of the gut environment, kinetics of resistance development with phage concentration dependency [21], resistance phenotype reversion [11,13] and selection for phase variable receptor structure leading to continuing co-evolution [12] must be taken into account when developing future phage strategies. One optimistic view is that, in contrast to antibiotic resistance, phage-resistance will be naturally kept under control via coevolution. In a longitudinal study on bacteria and phage interactions in a broiler house, Connerton et al. observed that although phage-resistant bacteria did emerge, they never outgrew and dominated the susceptible ones [68].

### 3.4. Vibrio Cholerae

Phages play a critical role in the evolution of pathogenic bacteria and especially that of *V. cholerae*, the causative agent of cholera epidemic diarrhoea. This is, for example, the case for the transmission of the cholera toxin into a nontoxigenic strain via integration of the lysogenic filamentous phage CTX_3 [69].

Phage-resistance appears to plays a central role in the evolution and regulation of this species in its natural environment. In a landmark three-year study in Bangladesh, Faruque et al. showed that the presence of phages infecting a given serogroup of *V. cholerae* was inversely correlated with the presence of viable *V. cholerae* of the same serogroup in the aquatic environment [70]. In addition, if a strain of a specific serogroup was observed in water samples with a phage infecting strains of the same serogroup, the strain was resistant to the coincidentally isolated phage in 73% of the case. During that period, the number of cholera patients correlated seasonally with the presence of *V. cholerae* in water samples devoid of cholera phages, and inter-epidemic periods correlated with water samples containing only cholera phages. These observations strongly confirmed the concept of fluctuating waves of different environmental *V. cholerae* serogroups existing in the aquatic environment, with successive rounds of phage amplification and selection of new resistant serogroups. The timing of phage peaks in the environment was also correlated with phage peaks in the stools of the patients, with increasing amount of phage particles in patients as the epidemic progressed [71]. In addition, phages excreted in cholera stools were the same as those found in the environment during the late stage of the epidemic and were expected to mediate *V. cholerae* elimination and antagonize its transmissibility. Indeed, *V. cholerae* populations recovered from phage-positive patient stools were significantly less infective than phage-negative stools in an animal model [72].

Interestingly, when phage-positive stools were cultured in rich nutrient medium, but not environmental water, a rapid emergence of phage-resistant variants that had lost the O1 antigen was observed. Since the O1 antigen is important for protection from the environmental stress and to escape host immune defences, it was suggested that the dominance of phage-resistant variants should not be able to sustain an ongoing epidemic. Indeed, phage-resistant *V. cholerae* variants having altered O1 antigens were significantly less able to colonize the small intestine of mice [33]. O1 antigen alteration was also observed to be phase variable due to single nucleotide deletions in two genes critical for O1 antigenic variation [32]. Indeed, modulation of O1 antigen in *V. cholerae* is important to escape O1 antigen specific phages in nature. Although O1 phage variants were attenuated in a mouse model of intestinal colonization, positive selection of revertants was shown in the intestinal tract. As a consequence, the intestinal environment favours O1 revertant that are infectious, but simultaneously susceptible to phages [32,72].

The phage content of patients’ stools was analysed during a 10-year survey in Dhaka, Bangladesh [73]. One phage, ICP1, was present in all stools from cholera patient and used the O1 antigen of lipopolysaccharide as receptor. This suggests that ICP1 is extremely well adapted to its host with a high selective pressure to maintain its genomic structure. Two other phages (ICP2 and ICP3) were only transiently observed. ICP2 and ICP3 are not O1-specific, which explains why they were less frequent, since *V. cholerae* O1 is the predominant serotype.

The surface receptor of phage ICP2 is the OmpU outer membrane protein. *V. cholerae* resistant variants with decreased OmpU expression were described, and had attenuated virulence in an infant mouse colonization model in vivo [74].

Since a cocktail composed of phage targeting different bacterial receptor would reduce the chance of phage bacterial multi-resistance, Yen et al. reasoned that a cocktail composed of the three different ICP phages could be used to prevent cholera infection [22]. The tree-ICP cocktail could kill *V. cholerae* in vitro and prevent intestine colonization or cholera-like diarrhoea of infant mice and rabbit models. All isolates from mice having received the phage cocktail 6 or 12 h after bacterial challenge were sensitive to all three phages. Resistance was, however, observed for mice having received the cocktail 24 h before bacterial infection, raising the question of phage partial washout prior to bacterial inoculation, and thus incomplete efficacy. These resistant variants had a mutation in the O antigen gene for phage ICP 1 and ICP 3 resistant variants, and OmpU for phage ICP 2 resistant variants. None of the isolates were resistant to all three phages.

### 3.5. Experimental Meningitis and Endocarditis

The intrinsic bactericidal properties of anti-infective compounds such as phages can be reliably studied in models where host defences are poorly involved. Such models of therapeutic sanctuaries include experimental meningitis and experimental endocarditis (EE). Experimental meningitis implicates a special anatomical setting where drug distribution depends on the blood–brain barrier. In contrast, EE mirrors the general situation encountered in many deep-seated infections where pathogens on the cardiac valves surround themselves with amorphous aggregates of platelet-fibrin clots, which cellular host defences cannot penetrate (for review see [14]). Thus, the capability of antimicrobials to cross the blood-brain barrier for meningitis or to penetrate into cardiac valve lesions (also called vegetations) are critical issues in these models.

Early studies by Smith and Huggins used a mouse model of meningitis. Mice were infected with *E. coli* 018:K1:H7ColV+ and treated 16 h later with one intramuscular dose of anti K phage or 12 doses of tetracycline, ampicillin, chloramphenicol, or a mixture of trimethoprim and sulfamethoxazole. The mortality was significantly lower in phage-treated mice than in the different antibiotics groups. Isolates recovered from mouse brains were tested for phage and antibiotic resistance. No antibiotic resistance was detected. In contrast, 6 out of 360 independent colonies (observed in 5/36 of the mice) recovered from phage-treated mice were phage resistant. All six phage-resistant isolates were K1 antigen negative, predicting decreased infectivity.

Oechslin et al. examined the efficacy of an antipseudomonal cocktail of 12 phages, used alone or in combination with antibiotics, in a dual in vitro and in vivo model of *P. aeruginosa* experimental endocarditis [24]. First, ex vivo fibrin-platelet clots were inoculated with 10^8^ log CFU of *P. aeruginosa*. Phage treatment rapidly decreased bacterial counts by 6 log CFU in 6 h. However, bacterial regrowth was observed after 24 h due to the selection of resistant variants. The rate of phage-resistance mutation in the original inoculum was of ca. 10^−7^, and resistant mutants expectedly took over after initial phage-induced killing, as described by Luria and Delbrück [45]. Bacterial regrowth after 6 h was prevented by the addition of sub-inhibitory concentrations of antibiotics, namely ciprofloxacin or meropenem.

In rats with experimental aortic endocarditis, phage therapy decreased vegetation bacterial counts by 2.3 to 3 log CFU, depending on the mode of administration. Phage therapy alone was comparable to ciprofloxacin. However, combining both treatments resulted in a highly synergistic effect with 7/11 (64%) of rats having culture-negative vegetations after only 6 h, an unprecedented efficacy in this very experimental setting.

Most importantly, phage-resistant *pseudomonas* variants were not observed in in vivo endocarditis therapy, either before or after treatment, therefore suggesting that phage-resistance could result in altered virulence or altered fitness of bacteria in animals. The hypothesis was investigated by characterizing two phage-resistant variants recovered from the ex vivo fibrin clot experiments, which displayed either transient resistance to all phages present in the cocktail or total resistance against 10 of the 12 phages. Total genomic sequencing and comparison disclosed that one of the variants had a 15 bp deletion in the *pilt* ATPase gene involved in pilus retraction, thus resulting in altered twitching motility. The other phage-resistant variant had lost the O-antigen and LPS core due to a large 350 kb deletion encompassing the *galU* gene, which is involved in LPS synthesis. Both resistant variants were less able to infect sterile vegetations than the parent strain. Since both pilus and LPS are virulence factors, it was concluded that mutations conferring phage-resistance come at a high physiological cost in fitness and virulence.

### 3.6. Sepsis and Acute Infections

Pouillot et al. evaluated phage therapy in a murine model of fatal neonatal sepsis [25]. Rat pups received intraperitoneal injections with a virulent strain of *E. coli* O25b:H4-ST131 and were treated 7 h or 24 h post infection with subcutaneous injections of monophage EC200^PP^. Phage therapy administered 7 h post infection rescued all the rats, whereas delaying therapy until 24 h rescued only 50% of the animals. Phage-resistant colonies with rough morphologies were recovered from the treatment failures. However, these variants were more susceptible to serum-induced killing and their virulence was dramatically attenuated in a sepsis model. Smith and Huggins made similar observations when injecting mice in one gastrocnemius muscle with *E. coli* 018:K1:H7 and injecting phages in the contralateral muscle [23]. Phages were efficient in decreasing bacterial muscle densities, and only very few phage-resistant variants were recovered at the inoculation site. These isolates were K1 antigen negative, which was previously shown to decrease virulence in mice [75].

Hung et al. used an experimental mouse model of *Klebsiella pneumoniae* liver abscess. Both intraperitoneal and intragastric administration of a single phage NK5 protected mice from death in a dose-dependent manner [26]. *K. pneumoniae*-induced liver injury and inflammatory cytokine production were significantly decreased by phage therapy. As in the experimental endocarditis study [24], phage-resistant variants emerged after 6 h or 12 h during phage time-kill curves in vitro, but no phage-resistant variants were observed in vivo. The resistant variants selected in vitro had lost the hypermucoviscosity characteristic of *K. pneumoniae* NK-5. Five individual resistant variants were tested for virulence in an intragastric model of infection and were significantly less virulent than the parent strain. The phage-resistant variants were more susceptible to phagocyte-induced killing. Gu et al. also observed the emergence of phage-resistant *K. pneumoniae* variants in vitro that were less virulent in vivo [29]. These variants exhibited colony morphology variations with a rough phenotype, as compared to the large and smooth wild-type colonies. This morphological feature of variant strains remained stable even after repeated subculture and storage at –80 °C. Variants also displayed much weaker virulence when intraperitoneally injected into mice.

Using a different type of model, Park et al. observed the effect of oral administration of phage-impregnated food (mixture of two different phages PPpW-3 and PPpW-4) to ayu fish infected by *Pseudomonas plecoglossicida* [28]. *P. plecoglossicida* were always detected in the kidneys of non-treated control fishes, while they were rapidly eradicated in fishes receiving phage therapy. Bacteria recovered from dying non-treated controls were susceptible to both phages. In contrast, phage-resistant variants were observed in liquid cultures after exposure to phages PPpW-3 and PPpW-4. Four individual variant isolates (three resistant to both phages and one resistant to phage PPpW-4 only) were tested in vivo by intramuscular injection. While the parent strain was highly virulent, all four resistant variants were avirulent, even at high inocula. In addition, one peculiar strain of *P. plecoglossicida*, which was highly virulent following intramuscular injection in ayu fish, was also tested and became poorly virulent after selection for phage-resistance. Moreover, bacterial growth in freshwater was observed to be lower in the presence of phages, and the number of phage PFUs increased rapidly, indicating phage predation and replication. These results are reminiscent of the *V. cholerae* phage ecology, and suggest that it might be possible to use phages to control *P. plecoglossicida*-induced disease in fish.

Finally, Lerodelle and Poutrel evaluated the potential of phage therapy to cure sub-clinical mastitis due to *Staphylococcus aureus* in lactating cows [27]. Udders were inoculated with *S. aureus* 106-6 and 107-59 via the mammary ducts and bacteriophage K lysates were administered by the same route once sub-clinical mastitis was confirmed. Phage therapy decreased *S. aureus* bacterial loads in 60% to 100% of the animals within 48 h of treatment, but could not sterilize all the udders. Treatment failures were attributed to the deep-seated and intracellular localization of *S. aureus* in mastitis, which hide bacteria from extracellular phages. Phage resistance was not responsible for treatment failure, as virtually no resistant variants were recovered.

## 4. Phage Resistant Variants for Vaccine Production and Studying Virulence Factors

The potential of phages to select for resistant variants with decreased in vivo virulence was used to generate vaccines against *S. enterica* or *S. aureus*. Capparelli et al. isolated a phage-resistant variant of *S. enterica* serovar Paratyphi B selected with phage φ1. The resistant variants formed smaller colonies and had lost their O-antigen; this phenotype was stable over many subculture passages. In addition, phage-resistance was also associated with impaired transcription of six virulence factors, resulting in an avirulent phenotype when inoculated intravenously into mice. Remarkably, immunization of mice with the resistant variant protected the animals against infection with the lethal parent strain, with 100% efficacy. As a control, vaccination with the heat-killed parent strain did not elicit protection.

The authors also observed that immunization of mice with phage-resistant *S. aureus* mutants conferred broad-spectrum immunity against this pathogen [35]. Acquisition of phage-resistance against phage M^SA^ resulted in several altered properties from the *S. aureus* parent strain, including teichoic acid alteration—which was responsible for resistance—reduced growth rate, decreased expression of several virulence factors, and increased production of capsular polysaccharides. All these features were stable during prolonged subculturing. Intramuscular administration of the phage-resistant variant protected mice from lethal doses of the wild-type parent strain in 90% of the animals.

Regarding virulence factor studies, Filippov et al. used site-directed mutagenesis of different LPS genes involved in the inner and outer core synthesis, followed by trans-complementation, to determine six *Yersinia pestis* phage receptors [36]. Phage-resistant mutants had attenuated virulence with increased LD50 and time-to-death in mice, including five mutants that became totally avirulent. Likewise, Lannto et al. reported loss of virulence driven by phage-resistant *Flavobacterium columnare* in a zebrafish model of infection [37]. Phage-resistant variants produced rough colony morphotypes and exhibited impaired gliding motility, a phenotype that was maintained over ten serial passages in liquid culture. Virulence of the parental morphotype was compared to the phage-resistant R type in a zebrafish infection model. The R type mutant became completely avirulent.

Heierson et al. reported that phage-resistant mutants of *Bacillus thuringiensis* had a decreased virulence phenotype in pupae of the Cecropia moths, which correlated with flagella loss and an increased susceptibility to methicillin [38]. In two studies performed by Flyg et al., phage-resistant variants of the insect pathogen *Serratia marcescens* were also observed to have decreased resistance to insect immunity and decreased virulence in a drosophila infection model, although the exact reason for this virulence decrease was not described [39,40]. Finally, Regeimbal et al. also observed that *Acinetobacter baumannii* phage-resistant variants that had lost their capsule became avirulent in a *Galleria mellonella* model [76].

While alterations of virulence features related to phage resistance might be useful for vaccination, they also help understand bacterial pathogenesis. In this regard, the above-mentioned studies support the fact that phage resistance may be accompanied by fitness costs for the bacteria that may benefit the host. As a result, the emergence of phage resistance during phage therapy is not always synonymous with treatment failure.

## 5. The Biological Cost of Antibiotic Resistance and the Combined Action of Phage and Antibiotics

As for phage–bacteria coevolution, antimicrobial resistance is an ancient process that results from the complex interaction between many microorganisms in their natural environment. Indeed, most antibiotics are naturally-produced toxic molecules against which bacteria had to evolve protective mechanisms in order to survive [77]. Antibiotic-resistant bacteria are an increasing problem in human and veterinary medicine, as well as in the farming industry, due to the overuse of antibiotics over the last half century. Antibiotic resistance may be intrinsic (i.e., bacteria may be naturally resistant to certain antibiotics) or may result from spontaneous mutations or from the acquisition of horizontally-transferred resistance genes [78]. Foreign gene acquisition may involve DNA transformation, cell-cell conjugation, and phage-mediated transduction.

Resistance mechanisms include structural alteration or decreased expression of the antibiotic target, decreased drug accumulation (via decreased permeability or increased drug efflux), or changes in global metabolic pathways (for review on the topic see [79]).

Since antibiotics target important physiological functions, such as protein synthesis, cell wall synthesis, or DNA replication, antibiotic resistance often implies a certain fitness cost (for a review on the topic see [80]). However, the fitness cost associated with resistance may sometimes become counterbalanced by compensatory mutations. This was the case in *E. coli*, were streptomycin resistance conferred by mutations in the ribosomal protein RpsL first decreased the speed of protein synthesis, but were compensated after several passages (evolved cultures) by neighbouring mutations that restored the speed of protein synthesis [81]. Likewise, acquisition of the tetracycline and chloramphenicol resistance plasmid pACYC184 by *E. coli* decreased its growth rate. The growth speed was recovered, and even surpassed in evolved cultures, thanks to adaptive mutations present on the bacterial chromosome (not on the plasmid), which took advantage of the tetracycline-resistance efflux pump [82]. Thus, it was the bacterium that took advantage of the presence of the plasmid, not the plasmid that took advantage of the bacterium. Numerous other examples of adaptive mutations exist both in vitro and in vivo [83,84]. In addition, the acquisition of mobile genetic elements can also lead to co-selection to more than one antibiotic resistance if different resistance genes are genetically linked [85], and such multi-resistance is not incompatible with the restoration of fitness, as exemplified in the pACYC184 experiments [82]. Therefore, as with phages, bacteria undergo dynamic evolutionary processes when challenged with antibiotics.

On the other hand, combining both phages and antibiotics could act in synergism to prevent resistance or increase therapeutic efficacy (for review on the topic see [86]). Verma et al. showed that combining ciprofloxacin and phages prevented the emergence of phage-resistant variants during treatment of *K. pneumoniae* biofilms, although no direct bactericidal synergism between phages and antibiotics was observed [87]. The emergence of phage-resistance was also prevented by treating *S. aureus* (in continuous culture) with a combination of phages and gentamicin [88]. Torres-Barcelo et al. confirmed the potential benefit of combining phages and antibiotics (in this case, streptomycin) against *P. aeruginosa* [89]. The phenomenon of phage-antibiotic synergism was also observed in animal experiments of *P. aeruginosa* endocarditis [24], as well as with the multi-resistant bacterium *Burkholderia cepacia* [90].

In addition, Chan et al. showed that selection of phage-resistance could also restore antibiotic susceptibility [91]. When using a lytic phage specifically targeting bacterial receptors that are part of the multidrug efflux systems, MexAB and MexXY—for instance, the outer membrane porin OprM—phage-resistance restored antibiotic susceptibility because the efflux pump, which confers resistance to several antibiotic classes, was no longer functional.

However, phage-antibiotic synergism may be dependent on experimental systems, and especially antibiotic dosages. Cairns et al. showed that using sub-inhibitory concentrations of streptomycin, as might be found in natural environments or sewage, could increase the rate of phage-resistance mutations in *Pseudomonas fluorescens*, and, conversely, phage exposure increased the rate of mutation to streptomycin resistance [92], which is compatible with phage-induced bacterial mutations described by Pal et al. [61]. Nevertheless, looking at the association between antibiotic and phage-resistance in a large collection of laboratory or clinical *E. coli* isolates, Allen et al. did not find a positive or systematic correlation between drug-resistance and phage-resistance, suggesting that antibiotics used in medicine or agriculture are unlikely to induce changes in phage resistance or phage-antibiotic cross-resistance in the environment [93].

Taken together, while different kinds of positive or negative phage–bacteria interactions can be observed in the laboratory or under natural conditions, potentially useful synergistic interactions do exist and could be valuable to use in specific clinical situations.

## 6. Phage-Resistance in the Setting of Phage Therapy

While the use of phages as therapeutic agents is conceptually simple, complex questions still exist regarding host range, route of administration, pharmacokinetic/pharmacodynamic parameters, and managing the risk of resistance. One of the main differences between phages and antibiotics is the ability of phages to self-replicate at the infection site. Therefore, the pharmacokinetics of phage therapy is closer to the population dynamics of predator-prey models described in co-evolutionary studies (see Section 1) than classical peak-distribution-elimination phases classically measured for antibiotics. Levin and Bull proposed theoretical predictions for modelling interactions between phages and bacteria during phage therapy of acute infections [94]. Assuming that there is a bacterial density threshold beyond which the patient dies, and a limit in host defences, below which bacterial growth cannot be controlled, the absence of therapy may lead to a situation where host defences cannot keep bacteria in check in order to prevent death. By combining host defence and phage therapy, the bacterial growth rate becomes negative before it reaches the lethal density threshold. In addition, the remaining host defences are likely to more easily hinder the delayed growth of phage-resistant bacteria before they reach the lethal threshold. In a recent study done by Roach et al., the effect of host immunity and phage-mediated bacterial clearance was investigated in a mouse model of acute *P. aeruginosa* pneumonia [31]. Phage therapy using healthy mice and mice with various immune defects revealed that neutrophil-phage synergism was essential for the resolution of disease. Indeed, phage therapy failed to prevent fatal outcomes in mice with neutrophil signalling defects due to the outgrowth of phage-resistant variants. In silico analysis also predicted that neutrophils were important to prevent the emergence of phage-resistant variants and to efficiently clear infection. Thus, without immune activation, phage-resistant mutants overwhelm the basal immune defences and lead to a resurgence of a phage-resistant population that ultimately causes mortality.

Two general models of phage therapy implementation were proposed in order to manage the risk of phage-resistance. First using phage cocktails and second adapting single phages to each patient condition, referred to as personalized phage therapy.

The main reason for combining multiple phages in cocktails is to broaden the phage host range and improve effectiveness by increasing the number of potential target pathogens. This results in a greater potential for empirical treatment [95,96]. Regarding the emergence of resistance, the different phages present in the cocktail are expected to synergize by targeting different receptors on the bacterial surface, resulting in a lower statistical chance of bacterial co-resistance, as with combined phage-antibiotic therapy. This was supported by Gu et al. who observed significantly lower frequencies of phage-resistant *K. pneumoniae* mutants using a cocktail of three phages, compared to monotherapy [29]. Similar observations were made with *E. coli*, where phage cocktails decreased the frequency of phage-resistance or delayed the emergence of phage-resistant variants [97,98]. This broad spectrum antimicrobial strategy is reminiscent of the model developed by pharmaceutical companies for antibiotics, with the risk of treatment failure in case of a lack of susceptible bacteria, as well as the risk of selecting resistance in fortuitous innocuous bacterial bystanders [96]. This approach is used in countries such as Georgia, where phage cocktails are administered as an empiric treatment, although the phage content may change over time in order to adapt to the most prevalent pathogens [99]. Alternatively, existing phages can also be adapted to existing phage-resistant strains [100].

The personalized phage strategy uses single phages or targeted phage cocktails directly formulated from a phage bank according to the pathogen isolated from the patient [96,100]. Although this strategy entails a higher cost associated with personalized treatment, it offers much more flexibility regarding the spectrum of the phage and can counter the emergence of bacterial resistance more efficiently. In one of the few well-documented phage therapy clinical trials that took the emergence of phage-resistance into account, Międzybrodzki et al. achieved ca. 40% of a positive clinical outcome with 20% pathogen eradication using phage monotherapy [101,102]. Following phage therapy, phage typing patterns of the pathogens were modified in 70% of the patients treated for *S. aureus* infection (53 patients in total), 91% for *P. aeruginosa* (11 patients in total), and 100% for *E. faecalis* (14 patients in total), and *E. coli* (14 patients in total). Resistance of the target pathogen to the therapeutic phage was also observed in up to 17% of *S. aureus* cases, 36% of *P. aeruginosa*, 43% of *E. faecalis*, and 86% of *E. coli*. The high frequency in the *E. coli* infection group was a cause of frequent change of the phage during the treatment. Complete resistance to any of the phages present in the phage collection of the Ludwik Hirszfeld Institute, Poland, was observed in 7% of the *S. aureus* cases, 27% of the *P. aeruginosa* cases, 21% *E. faecalis* cases, and 27% of the *E. coli* cases.

Emergence of resistance during phage therapy was also documented by Zhvania et al. in a recent case study of chronic *S. aureus* skin infection at the Eliava Phage Therapy Center, Georgia [103]. Treatment with two anti-staphylococcal products greatly improved the patients’ symptoms starting from seven days posttreatment. However, phage-resistance to the phage cocktail (Pyobacteriophage) was observed after three months of treatment and an alternate phage cocktail had to be substituted.

The use of personalized phage therapy was also exemplified in a case report by Schooley et al., where personalized-based therapeutic phages were administered parenterally to successfully treat one patient with a disseminated multidrug resistant *A. baumannii* infection. Different phage cocktails were assembled based on time-kill assays using a library of 96 phages. In vitro tests by serial passages revealed a stepwise selection of resistance to two of the cocktails. A third phage cocktail was prepared using the resistant isolates, which was then administered to the patient until the successful outcome of the infection. Of note, the phage-resistant phenotype that arose over time was associated with increased antibiotic susceptibility when phage and antibiotics were simultaneously administered. In addition, differences in colony morphology were observed during the therapy, with the eventual loss of the capsule. The authors speculate that the capsule loss may have contributed to the phage-antibiotic synergy, which included decreased virulence that had also been observed by the same author in previous studies [76]. In addition, another case of successful personalized phage therapy was reported in a lung transplant patient suffering from multi-drug resistant *P. aeruginosa* pneumonia [104]. Different phage cocktails were administered to the patient intravenously or by inhalation. Susceptibility of the bacteria to the phage was monitored during the treatment. New phage cocktails were administered as bacteriophage resistance emerged. As above, a shift in the antibiotic susceptibility pattern was also observed during phage treatment. Thus, while phage resistance does emerge, it is not prohibitive to phage therapy as long as it is carefully monitored in order to adapt the phage composition, and additional synergistic interactions with host defences or antibiotics may occur.

Finally, Khawaldeh et al. reported a successful case of adjunctive bacteriophage therapy for a refractory *P. aeruginosa* urinary tract infection [105]. The phage cocktail used for the study was composed of six lytic bacteriophages coming from existing bacteriophage libraries at the Eliava Institute in Tbilisi and were selected based on several isolates of the infecting *P. aeruginosa*. Bacteriophage counts was observed to remain high until after the disappearance of the target organism and then diminished sharply. Urine samples remained sterile for six months after the completion of antibacterial treatment and no bacteriophage-resistant bacteria arose during the time of the treatment.

## 7. Conclusions and Perspectives

Early studies suggested that phage–bacterial coevolution was limited to a few rounds of infection cycles. Resistance emerges following the selection of bacterial subpopulations carrying preexisting mutations and results in alterations in envelope determinants used by phages to adsorb on the bacterial surface. Hence, phage-resistant variants were totally immune to further infection and coevolution was rapidly stopped. These initial observations raised doubts regarding the use of phages as therapeutic agents because such rapid emergence of phage-resistance could hamper treatment effectiveness. However, phage-resistance is often balanced with resulting fitness costs for the bacteria. Indeed, abiotic/biotic factors, including environmental conditions, multiple bacterial exploiters, and resource availability, can greatly impact the successful emergence or stability of phage-resistance in natural environments.

The altered fitness of phage-resistant bacteria is believed to be important in phage therapy, where resistance mechanisms have been shown to alter virulence factors. In this literature review, the cost of phage-resistance was associated with virulence reduction in 17/22 (78%) of the articles (summarized in Table 1). Phage-resistant variants emerged in up to 82% of cases during phage-induced gut decolonization (out of 11 studies). Resistant variants were also reported after treatment of acute infection such as meningitis or sepsis, in up to 50% of the studies (out of six studies). Regarding the studied organisms, only 3/28 studies assessed the emergence of phage-resistance in Gram-positive bacteria, including two in *S. aureus* and one in *E. faecalis*. This focus on Gram-negative bacteria raises the question as to whether Gram-negative bacteria are more problematic regarding resistance selection.

Several lessons can be extrapolated from the reviewed studies. First, phage-resistant variants are often recovered after experimental therapy. Second, the intestinal milieu seems to be more prone to the evolution of phage-resistance, possibly due to its complexity, including mechanical viscosity and limited host defences in the lumen. Third, although phage-resistance often has a cost for the bacteria, it is not always associated with decreased infectivity, at least in the intestinal milieu.

From the five studies that clearly linked the emergence of resistance during phage therapy with alteration of a known virulence factor, like the O-antigen or LPS [14,15,22,23,24], four still reported resistant variants after therapy. The question then arises as to whether these variants were mere innocuous bystanders on the way of being eliminated by host defences, or whether they could still produce infection.

In any case, the ideal experimental setting should be to apply the Koch postulate and inoculate the variants to the animals in order to re-evaluate their infectivity. Indeed, recovering phage-resistant variants from in vivo samples may not be automatically synonymous with therapeutic failure, a counter-intuitive concept that appears to apply to phage therapy.

Regarding phage therapy clinical trials in human, the emergence of resistance seems to be a serious case of therapy failure if not monitored correctly. In three out of four clinical studies that monitored resistance, phage resistance led to adaptation of the composition of administered phages. Moreover, additional factors other than spontaneous mutations could also impact clinical resistance, including the immune status of the patient, the presence of biofilm, bacterial persistors, a chronic type of disease, and the possibility that the pathogenic strain possesses acquired types of resistance, like CRISPR [5,31,94,106].

It remains unclear whether the widespread use of phages to treat infections might lead to a problematic increase in phage-resistant bacterial pathogens in an analogous way that resistance developed to antibiotics. Although fitness cost may be associated with phage-resistance, this may depend on the environment, e.g., less virulence reduction associated in the intestinal milieu. Fitness was mainly assessed in the context of virulence, not in the bacterial survival in the environment. The initial fitness cost associated with antibiotic resistance could be compensated by adaptive mutations that stabilized resistant bacteria in the environment. In addition, as for antibiotics, horizontal transfer of phage resistance by plasmid acquisition was observed, which could be a problem in the long-term [107].

The real question is whether or not phages will be as widely used as antibiotics in the future. Antibiotics are used in medicine not only to prevent and combat infection, but also for other industrial applications in agriculture, which used up to 63,000 tons of antibiotics for livestock production alone in 2010 [108]. For now, it is more likely that phage therapy will be utilized as a more personalized medicine. In this case, the emergence of resistance will be manageable thanks to careful monitoring during therapy.

It is interesting to note that in the hypothetical emergence of a phage-resistant superbug, coevolution studies suggest that new phages will always be available in nature. Indeed, from environmental perspectives, bacteria were observed to be more resistant to their contemporary phages than to past or future phages and that hard-to-infect bacteria were infected by generalist phages and not specialists [63,109].

Finally, in addition of the use of phage particles themselves, or phage-antibiotic combinations, it is also possible to use purified phage lysins as a potential therapy. Recombinant phage lysins demonstrate high antibacterial activity, although they are mainly restricted to Gram-positive pathogens (reviewed in [110]). Regarding resistance, the lysin PlyG was evaluated for the possible resistance emergence after repeated treatment of *Bacillus anthracis* [111]. Spontaneous resistant mutants could not be detected, even when a compound like ethyl methanesulfonate was used to increase the bacterial mutation rate. This suggests that phage lysins target essential cell wall components that are unlikely to be modified by the host bacteria. Similar observations were made for *Streptococcus pneumoniae* and the phage lysin Pal, where repeated exposure to low concentrations of enzyme did not lead to resistant mutants [112]. More recently, Totté et al. successfully treated three cases of chronic dermatoses due to *S. aureus* with topical applications of the Staphefekt SA.100 endolysin product [113]. For all cases, resistance induction was not observed during long-term treatment, which is usually observed with antibiotic therapy.

The reviewed studies highlight both the potential power and the limits of phage therapy. Phages and bacteria are longstanding partners that have learned how to respect each other and coevolve together. The use of phages for therapy might be highly efficacious to eradicate pathogens in well-defined and circumscribed infected niches, particularly if used in combination with antibiotics. Their great advantage over antibiotics alone is their extremely rapid killing kinetics, which surpasses any known antimicrobial molecules, and the fact that they can self-replicate at the infection site. Other advantages are that they may increase antibiotic susceptibility in specific cases, and that the emergence of phage-resistant escape mutants may be prevented by antibiotics, or may carry alterations in virulence factors. These developments are promising, but should follow a thorough step-by-step developmental process, in order to avoid creating a resistance dead-end like that of antibiotics.

On the other hand, large scale or open field utilization of phage therapy, such as gut decolonization for the agricultural industry, is less certain. The coevolution dynamics of phage and bacteria is extremely sophisticated in such complex environments. Moreover, although the fascinating example of cholera control is inspiring, it is clear that phages never eradicated *V. cholerae*, whereas *V. cholerae* never got rid of the phage. As enlightening as this example may be, it primarily underlines coevolution, but not eradication, and not even efficacious population control, as epidemics still proceed.

## Figures and Tables

**Table 1:** Principal in vivo studies investigating the relation between phage therapy and phage resistance.

	Bacterium	Model	Phage Type	Treatment Outcome	Resistant Found in after Treatment?	Impact of Resistance on Virulence	Receptor	Ref.
Intestinal colonization	*Campylobacter jejuni*	Chicken intestinal colonization	CP8 and CP34	Bacterial decrease between 0.5 and 5 log10 CFU/g of caecal contents compared to untreated controls over a 5-day period post-administration.	Yes, at a freq. of 4%	Less infective at low dose. Rapid phenotypic reversion when reintroduced in chicken.	ND	[11,12]
	*Campylobacter jejuni*	Chicken intestinal colonization	phiCcoIBB35, phiCcoIBB37, and phiCcoIBB12	Phage cocktail decreases the titre of *C. jejuni* in faeces by approximately 2 log10 CFU/g when administered orally.	Yes, at a freq. of 13%	Not less infective. No phenotypic reversion when reintroduced in chicken.	ND	[13]
	*Escherichia coli*	Calf, piglet, lamb Ta diarrhoea	B44/1, B44/2, B44/3, P433/1, and P433/2	Protected calves against a potentially lethal infection, cured diarrhoea in piglets, improved the course of disease in lambs.	Most calves that did not respond to phage treatment had a high number of phage-resistant variants. No phage-resistant mutants were isolated from lambs.	Decreased virulence	Capsular polysaccharides	[14]
	*Escherichia coli*	Calf diarrhoea	B41/1	Rapid reduction of bacterial titres to numbers that are harmless.	Yes	Reduced virulence	Capsular polysaccharides	[15]
	*Escherichia coli*	Sheep, mouse, steer intestinal colonization	KH1 and SH1	Oral phage treatment did not decrease intestinal *E. coli* in sheep. Decreased the number of *E. coli* CFU in cattle. Phage therapy cleared the bacteria in a mouse model of intestinal *E. coli* O157 carriage.	No	-	ND	[16]
	*Escherichia coli*	Mouse intestinal colonization	T4 phage, oral	ND	Phage resistant bacterial strains dominated gut after 92 days.	ND	ND	[17]
	*Escherichia coli*	Mouse intestinal colonization	cocktail made of phages CLB_P1, CLB_P2, and CLB_P3	No bacterial level change in the faeces after treatment.	No	-	ND	[18]
	*Enterococcus faecalis*	Gnotobiotic mouse intestinal colonization	φ VPE25	Threefold drop in *E. faecalis* total intestinal load after 24 h of VPE25 treatment.	Phage resistant variant replaced WT during treatment.	Resistant variants can colonize intestine.	Integral membrane protein PIPEF	[19]
	*Salmonella enterica*	Chicken intestinal colonization	cocktail of phages, EP2, MUT3, M4, and YP	Significant difference between phage-treated and untreated groups.	Yes	ND	ND	[20]
	*Salmonella enterica*	Chicken intestinal colonization	φ10, φ25, and φ151	Phages reduced caecal colonization.	Phage-resistance occurred at a frequency commensurate with the titre of phage being administered.	Colonization levels of resistant variants in the ceca did not differ from the controls. Reversion observed after infection.	ND	[21]
	*Vibrio cholerae*	Infant mouse and rabbit cholera model	ICP1, ICP2, and ICP3	Oral administration of phages up to 24 h before *V. cholerae* challenge reduced colonization of the intestinal tract and prevented cholera-like diarrhoea.	Yes	Variants can colonize intestine.	O-Antigen	[22]
Meningitis	*Escherichia coli*	Mouse meningitis	phage R	One dose of phage was at least equivalent to multiple doses of antibiotics, whether administered intramuscularly or intrathecally.	Yes	Supposably reduced virulence as described in [14].	Capsular polysaccharides	[23]
Endocarditis	*Pseudomonas aeruginosa*	Rat infective endocarditis	cocktail made of phages 12 bacteriophages	3 log reduction or valve sterilisation when combined with antibiotics.	No	Reduced virulence	LPS and pilus	[24]
Sepsis	*Escherichia coli*	Rat neonatal sepsis	phage EC200^PP^	Phage administered 7 h postinfection rescued 100% of the animals and 50% after 24 h.	Phage resistant variant were found when phage treatment was delayed for 24 h.	Avirulence	ND	[25]
	*Klebsiella pneumoniae*	Mouse liver abscess and bacteraemia	Phage φNK5	Intraperitoneal and intragastric administration of phage 30 min after infection protected mice from death in a dose-dependent manner. Decreased bacterial burden and liver damage.	No	Reduced virulence	ND	[26]
	*Staphylococcus aureus*	Experimental cow mastitis	Bacteriophage K	Decreased bacterial load after treatment.	Yes	ND	ND	[27]
	*Pseudomonas plecoglossicida*	Fish haemorrhagic ascites	PPpW-3 and PPpW-4. Oral	Protective effects of phage treatment with lower and delayed mortality 1 or 24 h after bacterial challenge.	No	Reduced virulence	ND	[28]
	*Klebsiella pneumoniae*	Mice acute bacteraemia	GH-K1, GH-K2, and GH-K3	Phage cocktail significantly enhanced the protection of bacteremic mice against lethal infection.	ND	Reduced virulence	ND	[29]
	*Salmonella enterica Parathyphi B*	Mouse sepsis	phage φ1	Phage given concurrently with a lethal dose of bacteria rescued 100% of the animals.	ND	Avirulence	O-Antigen	[30]
pneumonia	*Pseudomonas aeruginosa*	Mouse acute pneumonia	PAK_P1	Treatment failed to prevent fatality due to subsequent bacterial outgrowth after 24 h in immunocompromised mice. 100% of bacteria recovered from phage-treated at 24 h were resistant.	Yes, in immunocompromised mice	ND	ND	[31]
Phage resistant variants for vaccine production and studying virulence factors	*Vibrio cholerae*	Infant mouse cholera model	ICP1	ND	ND	Attenuated in vivo.	O-Antigen	[32]
	*Vibrio cholerae*	Infant mouse cholera model	K139	ND	ND	Significantly reduced in its ability to colonize the mouse small intestine.	Core oligosaccharide	[33]
	*Vibrio cholerae*	Infant mouse cholera model	phage JA1	ND	ND	Impaired colonization	Capsule/O-antigen	[34]
	*Staphylococcus aureus*	Mouse vaccination	M^Sa^ phage	ND	ND	Avirulence	Teichoic acids	[35]
	*Yersinia pestis*	Mouse vaccination	L-413C, P2 vir1, φ JA1a, φ A1122, T7, T7Yp^e^, Pokrovskaya, Y, PST, R^h^	ND	ND	Atenuated or avirulent.	LPS	[36]
	*Flavobacterium columnare*	Zebrafish	FCL-1 and FCL-2	ND	ND	Avirulence	ND	[37]
	*Bacillus thuringiensis*	Cecropia moth	φ42, φ51,and φ64	ND	ND	Decreased virulence	ND	[38]
	*Serratia marcescens*	Cecropia moth, Drosophila	Phages φJ and φK	ND	ND	Decreased virulence	ND	[39]
	*Serratia marcescens*	Cecropia moth	Phages φJ	ND	ND	Decreased virulence	ND	[40]
